# Isolation of dental pulp stem cells with high osteogenic potential

**DOI:** 10.1186/s41232-017-0039-4

**Published:** 2017-04-10

**Authors:** Takazumi Yasui, Yo Mabuchi, Satoru Morikawa, Katsuhiro Onizawa, Chihiro Akazawa, Taneaki Nakagawa, Hideyuki Okano, Yumi Matsuzaki

**Affiliations:** 1 Department of Dentistry and Oral Surgery, Keio University School of Medicine, 35 Shinanomachi, Shinjuku-ku, Tokyo, 160-8582 Japan; 2 Department of Physiology, Keio University School of Medicine, 35 Shinanomachi, Shinjuku-ku, Tokyo, 160-8582 Japan; 3 Department of Dentistry and Oral Surgery, Kawasaki Municipal Kawasaki Hospital, 12-1 Shinkawadori, Kawasaki-ku, Kawasaki, Kanagawa 210-0013 Japan; 4 Department of Biochemistry and Biophysics, Graduate School of Health Care Sciences, Tokyo Medical and Dental University, 1-5-45 Yushima Bunkyo-ku, Tokyo, 113-8510 Japan; 5 Department of Cancer Biology, Faculty of Medicine, Shimane University, 89-1 Enya-cho, Izumo, Shimane 693-8501 Japan

**Keywords:** Bone regeneration, Dental pulp stem/progenitor cell, Flow cytometry, Isolation, Osteogenic potential, Low-affinity nerve growth factor receptor, THY-1, Transplantation, Cranio-maxillofacial

## Abstract

Dental pulp stem cells/progenitor cells (DPSCs) can be easily obtained and can have excellent proliferative and mineralization potentials. Therefore, many studies have investigated the isolation and bone formation of DPSCs. In most previous reports, human DPSCs were traditionally isolated by exploiting their ability to adhere to plastic tissue culture dishes. DPSCs isolated by plastic adherence are frequently contaminated by other cells, which limits the ability to investigate their basic biology and regenerative properties. Additionally, the proliferative and osteogenic potentials vary depending on the isolated cells. It is very difficult to obtain cells of a sufficient quality to elicit the required effect upon transplantation. Considering clinical applications, stem cells used for regenerative medicine need to be purified in order to increase the efficiency of bone regeneration, and a stable supply of these cells must be generated. Here, we review the purification of DPSCs and studies of cranio-maxillofacial bone regeneration using these cells. Additionally, we introduce the prospective isolation of DPSCs using specific cell surface markers: low-affinity nerve growth factor and thymocyte antigen 1.

## Background

Dental pulp, which contains connective tissue, mesenchymal cells, neural fibers, blood vessels, and lymphatics, is located at the center of the pulp chamber enclosed in mineralized dentin. The main functions of dental pulp are to produce dentin and to maintain the biological and physiological vitality of dentin [1]. Dental pulp stem cells/progenitor cells (DPSCs) in adult dental pulp tissue are induced to differentiate into odontoblasts to form reparative dentin in order to protect dental pulp [2, 3]. DPSCs and stem cells from human exfoliated deciduous teeth (SHEDs) have a high proliferative potential, an extensive self-renewal ability, and a multilineage differentiation capacity, with osteogenic, chondrogenic, adipogenic, neurogenic, and myogenic potentials [3–5]. In particular, DPSCs and SHEDs have a high mineralization potential and are considered to be useful in bone regenerative therapy [6–8]. Many studies regarding DPSCs have been reported because dental pulp tissue is easily obtained. In most previous reports, DPSCs were traditionally isolated by exploiting their ability to adhere to plastic tissue culture dishes [3]. However, adherent culture conditions on plastic dishes inevitably change the expression of surface markers and the biological properties of stem cells. Consequently, stem cell properties may diminish during adherent culture on plastic tissue culture dishes [9, 10]. Furthermore, DPSCs isolated based on their adherence to plastic are frequently contaminated by cells with different phenotypes. Additionally, the proliferative and osteogenic potentials vary depending on the isolated cells. It is very difficult to obtain cells of a sufficient quality to elicit the required effect upon transplantation. Considering clinical applications, stem cells used for regenerative medicine need to be purified in order to increase the efficiency of bone regeneration, and a stable supply of these cells must be generated. Here, we review the purification of DPSCs and the studies of cranio-maxillofacial bone regeneration using these cells. Additionally, we introduce the prospective isolation of DPSCs with high osteogenic potential.

## Bone regenerative therapy in the cranio-maxillofacial region

Bone regenerative therapies are required to treat many diseases affecting the cranio-maxillofacial region such as craniofacial abnormalities, bone defects following mandible tumor surgery, trauma, jaw bone necrosis, and bone augmentation for dental implants. Bone regeneration plays significant roles in the recovery of function and improvement of aesthetic disorders in the cranio-maxillofacial area. Autogenous bones harvested from the patient’s own body, such as the iliac bone, scapula, and fibula, have been used for major reconstruction of the maxillofacial area [11]. This bone grafting requires large-scale surgery, e.g., reconstruction using vascular pedicle bone grafts and particulate cancellous bone marrow with a titanium mesh [11, 12]. Autogenous bone from the chin and ramus of the mandible, allogenic bone, and xenogenic bone have been used for minor bone augmentation [13, 14].

Regenerative medicine studies have used various approaches such as osteoinductive chemical factors, osteoinductive growth factors, osteoinductive materials, extracellular matrix, and cell-based tissue engineering. Many studies of adult stem cell-based tissue engineering have sought to effectively regenerate bone in the maxillofacial area. One recent line of progress in stem cell research is bone regeneration using stem cells from bone marrow (BMMSCs). BMMSCs not only have high osteogenic and chondrogenic potentials, but also have an excellent regenerative potential to treat bone defects in vivo [15]. Therefore, these cells are considered to be very useful for bone regenerative therapies in the maxillofacial area. Several groups showed that tissue-engineered bone constructed with BMMSCs elicits beneficial effects in a mandibular defect model, a maxillary sinus floor elevation model, and a jaw malformation model [16–18]. In humans, injectable tissue-engineered bone formation using BMMSCs and platelet-rich plasma was applied to 14 cases for ridge augmentation and dental implant placement [19]. Furthermore, another group applied BMMSCs seeded onto β-tricalciumphosphate to upper jaw bone defects for dental implant placement after trauma [20].

Dental stem cells are an attractive option for regenerative therapy because they can be easily expanded to generate the number required for generation of graft materials. Furthermore, dental stem cells can be easily obtained in comparison with BMMSCs because exfoliated deciduous teeth and impacted third molar teeth are often extracted for clinical or orthodontic reasons. All dental stem cells (including DPSCs, SHEDs, periodontal ligament stem cells, dental follicle stem cells, and stem cells isolated from the apical papilla) are considered to be obtained via minimally invasive methods when isolated from these extracted teeth. They can give rise to proliferative cells and osteogenic cells under appropriate conditions [3, 4, 21–23].

## Characterization of stem cells from dental pulp

**Fig. 1:**
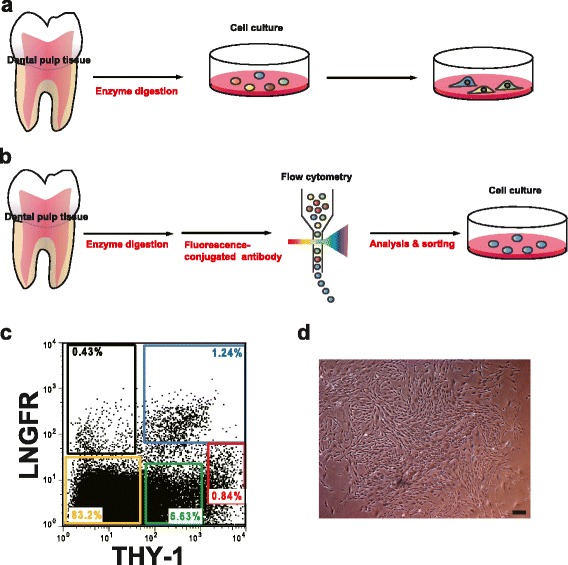
**a** Traditional isolation of dental pulp stem/progenitor cells (DPSCs) by adherent culture on dishes. **b** Prospective isolation of DPSCs by flow cytometric identification of cell surface markers. **c** Representative fluorescence-activated cell sorting profiles of dental pulp cells. **d** A representative phase-contrast micrograph of plastic-adherent colony-forming LNGFR^Low+^THY-1^High+^ cells with fibroblast morphologies. *Scale bars* = 100 μm

DPSCs are traditionally isolated from dental pulp by exploiting their ability to adhere to plastic tissue culture dishes after enzyme digestion [3] (Fig. 1a). This technique gives rise to heterogeneous cell populations that are frequently contaminated by other cells, including osteoblasts, osteoprogenitor cells, fat cells, reticular cells, macrophages, endothelial cells, and hematopoietic cells. There is a pressing need to enrich regenerative DPSCs. The study of DPSCs has been profoundly influenced by earlier studies of BMMSCs because DPSCs are positive for cell surface markers similar to those of BMMSCs, including CD44, CD73, CD105, STRO-1, and CD146, but are negative for CD45, CD34, CD14, C11b, CD79, CD19, and HLA-DR [5]. SHEDs also highly express MSC markers, including CD105, CD146, STRO-1, and CD29, but are negative for CD31 and CD34 [5]. Various methods have been tested to isolate and purify clonal subsets of stem cells from dental pulp, including immunoselection of cell surface markers by fluorescence-activated cell sorting (FACS) and magnetic-activated cell sorting (MACS) (Table 1).

**Table 1:** Purification of dental pulp stem/progenitor cells (DPSCs) and stem cells from human exfoliated deciduous teeth (SHEDs)

Authors	Year	Cell source	Enzyme digestion	Selection	Differentiation	Result
Shi et al. [24]	2003	Human DPSCs	3 mg/ml collagenase type I, 4 mg/ml dispase	STRO-1^+^ (MACS)	Odontogenic/osteogenic cells	Production of osteodentin-like structures and fibrous connective tissues
Laino et al. [27]	2006	Human DPSCs/SHEDs	3 mg/ml collagenase type I, 4 mg/ml dispase	c-Kit^+^CD34^+^STRO-1^+^CD45^−^ (FACS)	Osteogenic cells	High positivity for CD44, RUNX2, and osteocalcin
Iohara et al. [31]	2006	Human, bovine, canine, and porcine DPSCs	–	Hoechst 33342 (FACS)	Odontogenic, chondrogenic, adipogenic, and neurogenic cells	SP cells are enriched for stem cell properties and useful for cell therapy with BMP2 to regenerate dentin
Yang et al. [25]	2007	Rat DPSCs	–	STRO-1^+^ (FACS)	Odontogenic, neurogenic, adipogenic, myogenic, and chondrogenic cells	STRO-1 selection obtains a more homogeneous cell population with a multilineage differentiation capacity
Honda et al. [30]	2007	Human DPSCs	–	Hoechst 33342 (FACS)	Odontogenic cells	SP cells expressing ABCG2 in human adult dental pulp that differentiate into odontoblast-like cells
Waddington et al. [39]	2009	Rat DPSCs	4 mg/ml collagenase, 4 mg/ml dispase	LNGFR (MACS)	Osteogenic, adipogenic, \and chondrogenic cells	LNGFR^+^ DPSCs express CD105 and Notch 2
Ricco et al. [87]	2010	Human DPSCs	3 mg/ml collagenase type I, 4 mg/ml dispase	CD34^+^c-Kit^+^STRO-1^+^ (MACS)	Osteogenic cells	CD34^+^c-Kit^+^STRO-1^+^ DPSCs produce mineralized matrix in 2D and 3D cultures
Iohara et al. [33]	2011	Dog DPSCs	–	CD105 (FACS)	Odontogenic/osteogenic, adipogenic, angiogenic, and neurogenic cells	Transplantation of CD105^+^ DPSCs with SDF-1 completely regenerates dental pulp in a pulpectomy model
Mikami et al. [38]	2011	Human SHEDs	2 mg/ml collagenase type I	CD271^+^CD90^+^CD44 (FACS)	Osteogenic, adipogenic, chondrogenic, and myogenic cells	LNGFR positivity inhibits the differentiation of DPSCs into osteogenic, adipogenic, chondrogenic, and myogenic lineages
Hosoya et al. [58]	2012	Rat DPSCs	2 mg/ml collagenase, 0.25% trypsin	THY-1^High+^ (FACS)	Osteogenic cells	Hard tissue formation upon subcutaneous transplantation of THY-1^+^ cells
Kawanabe et al. [37]	2015	Human SHEDs	5 mg/ml collagenase type II, 2.5 mg/ml dispase I	SSEA-4^+^ (FACS)	Osteogenic, adipogenic, and chondrogenic cells	SSEA-4^+^ SHEDs have a multilineage potential.
Yasui et al. [50]	2016	Human DPSCs	2 mg/ml collagenase, 4 mg/ml dispase	LNGFR^Low+^THY-1^High+^ (FACS)	Osteogenic, adipogenic, and chondrogenic cells	LNGFR^Low+^THY-1^High+^ DPSCs promote osteogenic differentiation.

*BMP2* bone morphogenetic protein 2, *FACS* fluorescence-activated cell sorting, *LNGFR* low-affinity nerve growth factor, *MACS* magnetic-activated cell sorting, *SDF-1* stromal cell-derived factor-1, *SP* side population, *SSEA-4* stage-specific embryonic antigen-4, *THY-1* thymocyte antigen 1

DPSCs were first isolated from dental pulp tissue using cell surface markers, mainly STRO-1. Several studies reported that STRO-1^+^ cells have a high colony-forming ability and a multilineage differentiation capability [4, 24–26] and express CD146, and a pericyte marker (3G5) in perivascular and perineural sheath regions [24]. STRO-1^+^ and CD146^+^ cells in pulp of deciduous teeth are also located in perivascular regions [4]. c-Kit^+^CD34^+^CD45^−^ cells isolated from dental pulp by flow cytometry have a potent proliferative potential and readily differentiate into osteogenic precursors capable of generating three-dimensional woven bone tissue chips in vitro [27]. Although STRO-1^+^c-Kit^+^CD34^+^ human DPSCs (hDPSCs), which reside in a perivascular niche, have a lower proliferative capacity than STRO-1^+^c-Kit^+^CD34^−^ hDPSCs; they strongly express Nestin and the surface antigen low-affinity nerve growth factor (LNGFR, also called CD271) [28]. STRO-1^+^c-Kit^+^CD34^+^ hDPSCs show a stronger tendency toward neurogenic commitment than STRO-1^+^c-Kit^+^CD34^−^ hDPSCs, even though no significant differences between the two subpopulations arise after differentiation toward mesoderm lineages (osteogenic, adipogenic, and myogenic). c-Kit^+^FLK-1^+^CD34^+^STRO-1^+^ stem cells isolated from a plastic-adherent population by FACS have a potent growth potential (92% colony formation from 3–4 seeded cells) and are multipotent [9]. Other groups have demonstrated that colony-derived populations of DPSCs express typical mesenchymal markers, including CD29, CD44, CD90, CD166, and CD105 [29].

Subsequently, a side population (SP) was isolated from dental pulp based on efflux of the fluorescent dye Hoechst 33342 detected by FACS [30, 31]. This method, which has been used on SP cell populations from hematopoietic bone marrow, highly enriches cells with stem cell activity [32]. SP cells from dental pulp exhibit a self-renewal capacity with a long proliferative lifespan and differentiate into odontoblast-like cells, neurons, chondrocytes, and adipocytes [30, 31]. Furthermore, CD31^−^CD146^−^ SP cells and CD105^+^ cells from dental pulp have high proliferative and migration activities and a multilineage differentiation potential in vitro, including adipogenic, dentinogenic, angiogenic, and neurogenic potentials [33, 34]. In a whole dental pulp removal model, transplantation of canine CD31^−^CD146^−^ SP and CD105^+^ DPSCs expressing angiogenic and neurotrophic factors promotes regeneration of pulp in permanent teeth [33, 35]. Immature dental pulp stem cells express various embryonic stem cell markers [36]. A recent study of SHEDs demonstrated that stage-specific embryonic antigen-4^+^ cells derived from human deciduous dental pulp tissue have a multilineage differentiation potential in vitro [37].

Dental pulp originates from migrating neural crest cells; therefore, stem cells have been isolated from dental pulp using LNGFR, an embryonic neural crest marker [38, 39]. LNGFR has been used to prospectively isolate neural crest stem cells (NCSCs) from mammalian fetal peripheral nerves [40]. NCSCs can self-renew and differentiate into neurons, Schwann cells, and smooth muscle-like myofibroblasts in vitro. The characteristics of NCSCs are similar to those of MSCs. Cranial neural crest-derived cells contribute to ectomesenchymal cells in the developing dental papilla during tooth development [41, 42]. Cranial neural crest-derived LNGFR^+^ ectomesenchymal stem cells have odonto-differentiation potential [43]. Multipotent NCSCs have been identified not only in the early embryonic stage, but also in adulthood. Neural crest-related stem cells were isolated from mature dental pulp in several studies [39, 44, 45]. The enriched cell population expresses Nestin, LNGFR, and SOX10 and can be induced to differentiate into osteoblasts, melanocytes, and Schwann cells [45]. Thymocyte antigen 1 (THY-1, also called CD90)^+^ glial cells generate multipotent MSCs that produce dental pulp cells and odontoblasts [46]. LNGFR^+^THY-1^+^ neural crest-like cells derived from human pluripotent stem cells can differentiate into both mesenchymal and neural crest lineages [47]. Therefore, LNGFR and THY-1 could be useful to isolate clonogenic DPSCs from neural crest-derived dental pulp tissue.

## Prospective isolation of DPSCs using surface makers

Although many methods to enrich DPSCs have been devised, most assume that plastic-adherent cells are stem cells. Adherent culture on plastic dishes inevitably changes the expression of surface markers and gradually diminishes the differentiation, proliferation, and migration potencies of stem cells [9, 10]. These methods may not be able to reproduce the experimental results or reveal the biological properties of DPSCs. It is important to establish a method that can be used to prospectively isolate purified DPSC populations without cell culture. Therefore, specific cell surface markers need to be identified in order to isolate highly regenerative DPSCs. LNGFR and THY-1 have been identified as selective markers for the purification and phenotypic characterization of MSCs from various sources such as bone marrow, decidua, adipose tissue, and synovium [48, 49]. Especially in human bone marrow, LNGFR^+^THY-1^+^ cells are extremely enriched with clonogenic cells (2 × 10^5^-fold enrichment vs. whole bone marrow cells) [48]. Our study demonstrated that these markers can also be used to prospectively isolate hDPSC populations, thereby avoiding the need for prolonged cell culture [50] (Fig. 1b). Flow cytometric analyses revealed five cell populations, namely, LNGFR^+^THY-1^+^, LNGFR^Low+^THY-1^High+^, LNGFR^−^THY-1^Low+^, LNGFR^+^THY-1^−^, and LNGFR^−^THY-1^−^ (Fig. 1c). Although LNGFR^+^THY-1^+^ cells in bone marrow exhibit the highest clonogenic potential [48], assessment of the number of colonies showed that LNGFR^Low+^THY-1^High+^ cells in dental pulp have a significantly higher colony-forming potential than LNGFR^+^THY-1^+^ cells [50]. LNGFR^Low+^THY-1^High+^ cells are uniformly small and have a spindle-shaped (MSC-like) morphology (Fig. 1d). The cell population considered to be DPSCs comprises two cell types, and it seems that purity can be increased by selecting one of these. However, a LNGFR^Low+^THY-1^High+^ cell population was not observed in FACS profiles of human BMMSCs stained with anti-LNGFR and anti-THY-1 antibodies [48]. The discrepancy of the expression pattern of cell surface markers between dental pulp tissue and bone marrow tissue may be due to differences in the origin of the cells. Dental pulp tissue is thought to be derived from migrating neural crest cells, whereas bone marrow tissue originates from the mesoderm and neural crest [51, 52]. During development, neural crest cells from the dorsal neural tube migrate to various locations and divide into four main functional domains, namely, the cranial neural crest, the trunk neural crest, the vagal and sacral neural crest, and the cardiac neural crest. Neural crest cells differentiate into a vast range of cells, including neurons and glial cells of the peripheral nervous system, smooth muscle cells, bone, and cartilage cells. Each distinct cell type responds to specific migration and differentiation signals to generate the appropriate cells and tissues [53]. Therefore, the phenotypes and biological properties of each cell type may differ.

## Biological properties of stem cells from dental pulp

**Fig. 2:**
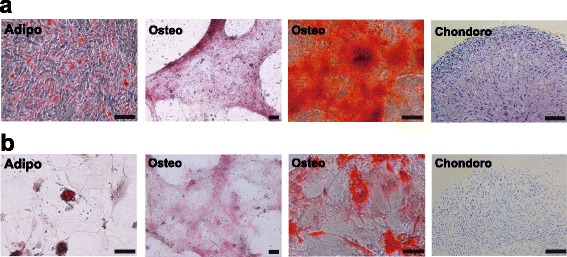
**a** Adipogenic (Adipo), osteogenic (Osteo), and chondorogenic (Chondro) differentiation of LNGFR^Low+^THY-1^High+^ cells. *Scale bars* = 100 μm. **b** Adipogenic (Adipo), osteogenic (Osteo), and chondorogenic (Chondro) differentiation of LNGFR^+^THY-1^+^ cells. *Scale bars* = 100 μm

DPSCs and SHEDs have a high proliferation rate and a multilineage differentiation capability, including osteogenic, chondrogenic, adipogenic, neurogenic, and myogenic potentials [3–5]. Osteogenic differentiation of DPSCs is easily induced in vitro by adding dexamethasone, ascorbic acid, and β-glycerophosphate to culture medium supplemented with fetal bovine serum [54, 55]. DPSCs express bone markers such as alkaline phosphatase, type 1 collagen, osteocalcin, and osteonectin under osteogenic induction [3, 56]. DPSCs have a faster population doubling time and a higher mineralization potential than BMMSCs [6, 7]. SHEDs have a higher proliferation rate and a higher capability for osteogenic differentiation than BMMSCs and even DPSCs [4, 57]. Overall, DPSCs and SHEDs are more suitable than BMMSCs for mineralized tissue regeneration. In our study, prospectively isolated LNGFR^Low+^THY-1^High+^ DPSCs showed a high clonogenic potential and a multipotent differentiation capability for mesenchymal lineages (Fig. 2a). The adipogenic, osteogenic, and chondrogenic capacities of LNGFR^Low+^THY-1^High+^ cells were higher than those of LNGFR^+^THY-1^+^ cells (Fig. 2a, b) [50]. Interestingly, the proliferation rates of LNGFR^Low+^THY-1^High+^ cells and LNGFR^+^THY-1^+^ cells did not significantly differ at early passages. Therefore, cultured hDPSCs isolated from crude dental pulp cells contain two cell types that originate from LNGFR^Low+^THY-1^High+^ and LNGFR^+^THY-1^+^ cells. High LNGFR expression may inhibit differentiation of hDPSCs into osteoblasts and adipocytes [38], while low LNGFR expression might maintain the stemness of hDPSCs in the dental pulp microenvironment. THY-1^+^ dental pulp cells localized in the sub-odontoblastic layer can differentiate into hard tissue-forming cells and may thus provide a source of odontoblastic cells [58]. THY-1^+^ human adipose-derived stromal cells show osteogenic potential in vitro and significantly increase bone formation in a calvarial defect model [59]. THY-1^+^ cells in other tissues also show a high proliferative capacity and osteogenic potential [60, 61]. These reports suggest that THY-1 is important to isolate stem cell-like cells with a potent mineralization potential. LNGFR^Low+^THY-1^High+^ DPSCs display a high proliferation rate and a long-term survival using a transillumination procedure such as cranial windows when transplanted into cranial defects of immunodeficient mice [50]. Therefore, LNGFR^Low+^THY-1^High +^ cells can increase the cell viability in cell transplantation, and this is considered to be advantage for differentiation into osteoblasts and secretion of each growth factor to promote bone morphogenesis. For successful tissue engineering, formation of blood vessels toward the transplanted tissue is required for transportation of oxygen and nutrients to the transplanted cells. When transplanted, stem cells such as DPSCs promote angiogenesis for bone regeneration in the maxillofacial region. DPSCs have a paracrine effect by stimulating the formation of blood vessels in the host tissue through secretion of angiogenic factors [62–68]. Furthermore, DPSCs and SHEDs may have stronger immunomodulatory properties and high anti-apoptotic activity [69–76]. Thus, DPSCs and SHEDs could also have potential for clinical applications in autologous stem cell transplantation for bone regenerative therapy.

## Studies of bone regeneration in the cranio-maxillofacial region using stem cells from dental pulp

There are many studies of bone regeneration using DPSCs and SHEDs in the cranio-maxillofacial region in vivo because these cells have high osteogenic potential (Table 2). Several studies reported that transplantation of expanded DPSCs and SHEDs with scaffolds, such as fibroin, collagen membrane, and hydroxyapatite/tricalcium phosphate ceramic particles, repairs critical-size cranial bone defects of mice and rats [8, 77, 78]. Yamada et al. demonstrated that cell-based therapy using stem cells derived from deciduous teeth and dental pulp of puppies together with platelet-rich plasma can induce new bone formation in critical-size mandibular bone defects [79]. Ito et al. demonstrated that the high osteogenic ability of DPSCs contributes to the osseointegration of dental implants [80]. Alkaisi et al. reported that SHEDs can enhance bone consolidation in a rabbit mandibular distraction model [81]. A study of a large animal model showed that stem cells from deciduous teeth of miniature pigs regenerate bone to repair critical-size swine mandible bone defects [82]. In terms of clinical applications of DPSCs in humans, a biocomplex constructed from DPSCs and a collagen sponge scaffold was reported to be useful for bone tissue repair in human mandibular bone defects after extraction of third molars [83]. However, these cells might have been contaminated by non-regenerative cells with a poor bone-formation ability because these studies did not use purified cells.

**Table 2:** Studies of bone regeneration by stem cells from dental pulp in the cranio-maxillofacial region in vivo

Authors and year		Targeted site	Cell source	Selection	Host	Scaffolds	Results
de Mendonca et al. [78]	2008	Cranial bone defect	Human DPSCs	–	Rat	Collagen membrane	Induction of mature bone formation
Seo et al. [8]	2008	Critical-size calvarial bone defect	Human SHEDs	–	Mouse	HA/TCP	Repair of defects and substantial bone formation
Zheng et al. [82]	2009	Orofacial bone defects	Stem cells from porcine (miniature pig) deciduous teeth	–	Miniature pig	β-TCP	More efficient regeneration of critical-size mandibular bone defects
d’Aquino et al. [83]	2009	Alveolar bone defect after extraction of impacted third molars	Human DPSCs	–	Human	Collagen sponge	Complete restoration of bone defects
Ito et al. [80]	2011	Osseointegration of dental implants	Canine DPSCs	–	Dog	PRP	High osteogenic potential to assist dental implant integration
Yamada et al. [79]	2011	Mandibular bone defect	Canine DPSCs and stem cells from deciduous teeth	–	Dog	PRP	Well-formed mature bone using both cell lines
Liu et al. [55]	2011	Critical-size alveolar bone defect	Rabbit DPSCs	–	Rabbit	rhBMP2 + nHAC/PL	Early mineralization and excellent bone formation
Ricco et al. [86]	2012	Critical-size cranial bone defect	Human DPSCs	CD34^+^c-Kit^+^STRO-1^+^ (MACS)	Rat	Fibroin scaffolds	Mature bone formation and defect correction
Pisciotta et al. [84]	2012	Critical-size parietal bone defect	Human DPSCs	STRO-1^+^ (MACS)	Rat	Collagen constructs	Restoration of critical parietal bone defects
Alkaisi et al. [81]	2013	Distracted area of mandibular bone	Human SHEDs	–	Rabbit	–	Enhancement of the bone consolidation period in mandibular distraction osteogenesis
Annibali et al. [77]	2013	Critical-size calvarial bone defect	Human DPSCs/PeSCs	–	Mouse	Porcine collagen + GDPB, β-TCP, Aga/nHA	β-TCP alone is more effective than β-TCP seeded with DPSCs/PeSCs
Giuliani et al. [85]	2013	Mandibular bone defect after tooth extraction	Human DPSCs	CD34^+^ (MACS)	Human	Collagen sponge	Regeneration of compact-type bone with uniform vascularization
Yasui et al. [50]	2016	Critical-size calvarial bone defect	Human DPSCs	LNGFR^Low+^THY-1^High+^ (FACS)	Mouse	Collagen membrane	LNGFR^Low+^/THY-1^High+^ DPSCs promote new bone formation to repair critical-size calvarial defects

*Aga/nHA* a sponge of agarose and nanohydroxyapatite, *DPSCs* dental pulp stem/progenitor cells, *FACS* fluorescence-activated cell sorting, *GDPB* granular deproteinized bovine bone, *HA* hydroxyapatite, *LNGFR* low-affinity nerve growth factor, *MACS* magnetic-activated cell sorting, *nHAC/PLA* nanohydroxyapatite/collagen/poly(L-lactide), *PeSCs* periosteal stem cells, *PRP* platelet-rich plasma, *rhBMP-2* recombinant human bone morphogenetic protein 2, *SHEDs* stem cells from human exfoliated deciduous teeth, *TCP* tricalcium phosphate, *THY-1* thymocyte antigen 1

Several studies investigated bone formation using hDPSCs purified by MACS for the repair of bone defects. Pisciotta et al. reported that STRO-1^+^ hDPSCs cultured in human serum-containing medium repair critical-size parietal bone defects in immunocompromised rats [84]. Giuliani et al. reported that CD34^+^ hDPSCs together with a collagen sponge regenerate compact bone with uniform vascularization after tooth extraction [85]. Ricco et al. reported that CD34^+^c-kit^+^STRO-1^+^ hDPSCs with fibroin scaffolds induce mature bone formation and repair critical-size bone defects in immunocompromised rats [86].

In our study, LNGFR^Low+^THY-1^High+^ and LNGFR^+^THY-1^+^ cells prospectively isolated by FACS were transplanted into critical-sized calvarial defects to evaluate their therapeutic potential [50]. LNGFR^Low+^THY-1^High+^ hDPSCs exhibit long-term survival and osteoblastic differentiation in immunohistochemical analyses. Microcomputed tomography-guided morphometric analysis showed that LNGFR^Low+^THY-1^High+^ cells induce the highest level of bone regeneration after transplantation into calvarial defects. The bone-formation potential of LNGFR^Low+^THY-1^High+^ cells is markedly higher than that of LNGFR^+^THY-1^+^ cells. Therefore, traditionally cultured DPSCs isolated from crude dental pulp cells are considered to comprise two cell types, namely, highly osteogenic cells and lowly osteogenic cells. We believe that enrichment of regenerative cells will lead to successful bone regenerative therapy through high levels of engraftment, survival, and proliferation post-transplantation.

## Conclusions

Considering clinical applications for bone regeneration, cell-based therapy using DPSCs requires a prolonged period of culture to obtain a sufficient number of cells for transplantation because only a small number of DPSCs can be obtained from a single tooth. Therefore, it is important to stabilize the quality and quantity of transplanted cells by ensuring they have high proliferative and osteogenic capabilities. Cultured DPSCs isolated from crude dental pulp cells are considered to comprise two cell types: regenerative and non-regenerative cells. Hence, isolation of the optimal cell population for bone regeneration is important for regenerative therapy. There is a pressing need to identify selective markers of DPSCs with high osteogenic potential. LNGFR and THY-1 can be used to prospectively isolate a pure population of DPSCs from human dental pulp by FACS. However, purification of DPSCs using these markers is still insufficient compared with that of BMMSCs. Consequently, it is necessary to further enhance their purity by using additional markers. Furthermore, specific markers of other easily obtained dental stem cells should be identified to acquire a cell source for cranio-maxillofacial bone regeneration in a future study because DPSCs cannot be obtained from non-vital teeth.

## Abbreviations

BMMSCs: Stem cells from bone marrow
CM: Conditioned media
DPSCs: Dental pulp stem cells/progenitor cells
FACS: Fluorescence-activated cell sorting
hDPSCs: Human dental pulp stem cells/progenitor cells
LNGFR: Low-affinity nerve growth factor
MACS: Magnetic-activated cell sorting
MSCs: Mesenchymal stem cells
NCSCs: Neural crest stem cells
SHEDs: Stem cells from human exfoliated deciduous teeth
SP: Side population
THY-1: Thymocyte antigen 1
